# Chitosan-Enclosed Menadione Sodium Bisulfite as an Environmentally Friendly Alternative to Enhance Biostimulant Properties against Drought

**DOI:** 10.1021/acs.jafc.2c07927

**Published:** 2023-02-09

**Authors:** David Jiménez-Arias, Sebastian Bonardd, Sarai Morales-Sierra, Miguel Â. Almeida Pinheiro de Carvalho, David Díaz Díaz

**Affiliations:** † ISOPlexis, Center for Sustainable Agriculture and Food Technology, Madeira University, Campus Universitário da Penteada, 9020-105 Funchal, Madeira, Portugal; ‡ Departamento de Química Orgánica, Universidad de la Laguna, Avda. Astrofísico Francisco Sánchez 3, La Laguna 38206, Tenerife, Spain; § Instituto Universitario de Bio-Orgánica Antonio González, Universidad de la Laguna, Avda. Astrofísico Francisco Sánchez 2, La Laguna 38206, Tenerife, Spain; ∥ Grupo de Biología Vegetal Aplicada, Departamento de Botánica, Ecología y Fisiología Vegetal-Facultad de Farmacia, Universidad de la Laguna, Avenida. Astrofísico Francisco Sánchez s/n, La Laguna 38071, Tenerife, Canary Islands, Spain; ⊥ CiTAB, Centre for the Research and Technology of Agroenvironmental and Biological Sciences, University of Trás-os-Montes and Alto Douro, Quinta dos Prados, 5000-801 Vila Real, Portugal; # Institute of Organic Chemistry, Faculty of Chemistry and Pharmacy, Regensburg University, Regensburg 93053, Germany

**Keywords:** menadione sodium bisulfite, nanoparticles, water deficit, biostimulants, nanoencapsulation

## Abstract

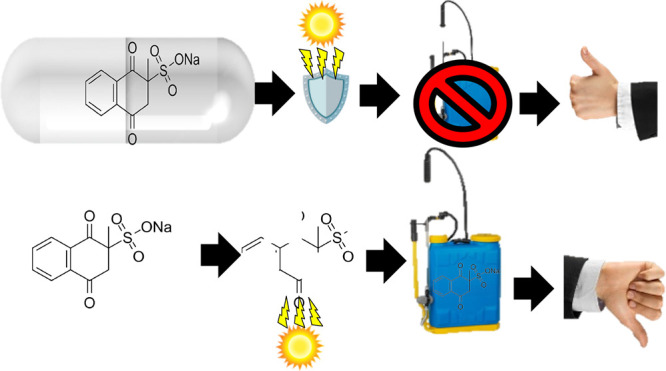

Biostimulants are an interesting strategy to increase crop tolerance to water deficits, and there is an extensive bibliography on them. However, most of them need to be treated continuously to increase protection throughout the growth cycle. In this context, we chose menadione sodium bisulfite, whose protective effect against water deficit has been previously demonstrated but only for a short period of time. Nanoencapsulation seems to be an interesting way to improve the properties of biostimulants. Our results show that menadione sodium bisulfite (MSB) encapsulated in chitosan/tripolyphosphate nanoparticles can increase the system’s tolerance against an imposed water deficit and delay the need for retreatment by at least 1 week, accelerating plant recovery after rehydration. This highlights the positive properties of nanoencapsulation and shows how a simple encapsulation process can significantly improve the biostimulant protective properties, opening up new possibilities to be explored under field conditions to cope with water-deficit stress.

## 1 Introduction

Environmental or abiotic stresses such as extreme temperature (heat or cold), salinity, nutrient deficit, flood, or drought account for the majority of worldwide agricultural losses.^1^ The latter is considered the greatest risk to crop production, which reached an estimated loss of $124 billion between 1998 and 2017.^2^ Droughts are occurring more frequently and more severely^3^ and are affecting the global economy.^4^ It is interesting to note that irrigation in agriculture accounts for 70% of global water use, even over 40% in many OECD countries,^5^ and the risk of yield losses is expected to increase due to climate change,^6^ thereby threatening future food security.^7^

Plants have evolved to cope with environmental stress. Due to their sessile nature, they have developed different coping strategies. The result is plants tolerant to excessive light, salt, temperature, and water deficit.^8^ Plants can adapt to survive under water-deficit conditions. Inadequate water status leads to various biochemical and physiological responses in plants, including a reduction in gas exchange parameters by closing stomata to prevent water loss.^9^ The accumulation of various compatible osmolytes, such as various sugars, sugar alcohols, betaines, and proline, helps to counteract osmotic pressure.^10^ The latter is widely used by plants to adjust the osmotic pressure created by water withdrawal.^11^

Moreover, plant defense mechanisms can be activated by external stimuli to accelerate stress acclimation, the most promising of which for crop production is the utilization of biostimulants.^12^ A biostimulant (Bs) is “a product stimulating plant nutrition processes independently of the product’s nutrient content, with the aim of improving one or more of the following characteristics of the plant: nutrient use efficiency, tolerance to abiotic stress, crop quality traits or availability of confined nutrients in the soil and rhizosphere”*.*^13^ These abilities of types of compounds to counteract abiotic stress are discussed in detail in the bibliography.^14^ Additionally, Bs is an economic hotspot and the global market is expected to reach $4.14 billion by 2025.^14^

An interesting compound capable of activating plant defense mechanisms is menadione sodium bisulfite (MSB), which is able to increase tolerance to abiotic and biotic stresses.^15^ It is interesting to note that, under a water deficit, if treated with MSB, plants show one of the best recovery responses to stress within the first week in comparison with other Bs,^16^ but the effect disappears in the second week. This behavior, in our opinion, is due to the sensitivity of MSB to changes in the environment, particularly light and pH changes.^17^ In addition, the use of pure bioactive compounds is very limited due to their rapid release, low solubility, and poor bioavailability.^18^ In general, greater than 90% of agrochemicals degrade during application, leading to economic losses and serious environmental hazards.^19^ An interesting solution is nanoencapsulation (NE).^20^

While NE in agriculture is relatively new and still in the early stages of development,^21^ there are examples of NE applications for fertilizer and pesticides for agricultural purposes.^22^ However, studies using Bs are practically residual.^23^ Among the wide variety of encapsulating polymers, chitosan (CHI) has shown to be a promising candidate due to its well-known biocompatibility and biodegradability, properties that have positioned this polysaccharide as a promising eco-friendly candidate to be used in agriculture.^24^ In this context, CHI is readily absorbed by plant surfaces (e.g., leaves and stems), extending the contact time between entrapped substances and plant tissue. In addition, CHI nanoparticles (Nps) facilitate passage through the cell membrane.^25^ Furthermore, these properties improve the molecular bioavailability of the active substances contained in the Nps.^24^ Furthermore, CHI and sodium tripolyphosphate Nps^26^ have proven the efficacy in the control of the release of salicylic acid during a 7 day period. Another interesting quality is the enhancement of crop productivity through encapsulation. Indeed, Nps offer an overwhelming opportunity to improve biostimulant application for agricultural usage.

Here, we report CHI Nps that enclose MSB, and subsequent plant responses to water deficit and rehydration are discussed to demonstrate how the reported Nps enhance MSB properties.

## 2 Materials and Methods

### 2.1 Materials

Low molecular weight chitosan with an acetylation degree of ∼25% (CHI, Sigma-Aldrich), sodium tripolyphosphate (TPP, 85%, Sigma-Aldrich), glacial acetic acid (AcOH, >99%, Sigma-Aldrich), sodium hydroxide (NaOH, ≥98%, Sigma-Aldrich), and menadione sodium bisulfite (MSB, ≥95% Sigma-Aldrich) were used. Ultrapure water was used throughout this study.

### 2.2 Preparation of MSB Chitosan/TPP Nanoparticles

MSB-loaded Nps were prepared based on previously reported protocols, which were adjusted considering our requirements.^27−29^ First, a 0.2% w/v CHI solution was prepared using as a solvent an aqueous solution of glacial acetic acid (0.6% v/v). This solution was stirred at room temperature overnight, followed by adjusting its pH to a value of 4.7 employing a NaOH aqueous solution (20% w/v) and passing through a 0.44 μm filter. On the other hand, a second solution was prepared by dissolving TPP into ultrapure Milli-Q water, achieving a concentration of 0.5 mg/mL. This solution was stored at 4 °C before use. Last, 2.64 mg of MSB was dissolved in 10 mL of the CHI solution and stirred (750 rpm) at 50 °C during 10 min, after which 6 mL of the cooled TPP solution was added dropwise and the mixture was rapidly transferred to an ice-water bath, maintaining the stirring conditions for 30 min. The volume of the obtained opalescent mixture was adjusted, achieving an MSB concentration of 0.6 mM, and stored at 4 °C. The same protocol was employed for the preparation of empty CHI Nps but without the MSB addition. The size and Z-potential values of MSB-loaded Nps were measured in triplicate using a Zetasizer Nano ZS (Malvern Instruments) particle size analyzer, yielding entities with an average size of 220 nm and a Z-potential value of +173.3 mV (Supporting Information).

### 2.3 Plant Material Experimental Conditions

*Solanum lycopersicum* L. was obtained from a local nursery vendor. Sowing in trays with a commercial substrate was done using an automatic seeder to ensure uniform germination and growth up to the two true leaf stage (BBCH-scale 12). The seedlings had 150 trays with a 3.5 cm-long and 3.5 cm-wide cell with a depth of 7 cm. Only well-rooted and disease-free seedlings of the same size were used for experiments. The seedling trays were placed in a growth chamber with controlled conditions: temperature, 24 ± 2 °C; photoperiod, 16–8 h (light/dark); humidity, 60–75%; irradiance, 300 μmol m^–2^ s^–1^.

### 2.4 Treatments and Water-Deficit and Rehydration Growth Assay

Water-deficit growth experiments were conducted over 7 days, following the procedure described by Jiménez-Arias et al.^30^ Stress was caused by irrigating with 50% less water with a half-strength Hoagland solution compared to control plants irrigated at full field capacity. This was repeated in all plants exposed to drought. After 7 days of water deficit, the rehydration trials started by fully irrigating all plants again for 7 days.

Plants were treated directly at the root with 1 mL of each treatment (Table 1) on the first day of the trial. After 2 h, the treated plants were watered with a half-strength Hoagland solution, 9 mL for well-watered plants and 4 mL for plants exposed to a water deficit. Experiments were carried out using eight different treatments as follows: WW, well-watered plants; N-WW, well-watered plants treated with empty nanoparticles; M-WW, well-watered plants treated with MSB at 0.6 mM; Mn-WW, well-watered plants treated with MSB-loaded Nps; WD, water-deficit plants; N-WD, water-deficit plants treated with empty nanoparticles; M-WD, water-deficit plants treated with MSB at 0.6 mM; Mn-WD, water-deficit plants treated with MSB-loaded Nps (Table 1).

**Table 1:** Treatments and Abbreviations Used in the Experiments

treatment	well-watered	water-deficit
no compound	WW	WD
MSB^a^	M-WW	M-WD
empty nanoparticle	N-WW	N-WD
MSB-loaded Nps	Mn-WW	Mn-WD

a In rehydration assays, a second treatment of MSB was performed on the first day called MSB_2_.

### 2.5 Growth Measures and Stress Index Calculations

For growth measures, 15 seedlings from each treatment were collected after 4 or 7 days of drought and at the end of rehydration. The plants were completely dried in an oven at 60 °C for 2 days and weighed separately. Different indices were calculated such as stress susceptibility index (SSI), stress tolerance index (TSI), relative growth rate (RGR), plant water use efficiency (WUE_p_), and relative water content (RWC) (Table 2).

**Table 2:** Stress Index Used in the Experiments^a^

index	formula
stress susceptibility index	SSI = (1 – (Dws/Dwp)/SII
stress intensity index	SII = 1 – (Dw̅s/Dw̅p)
tolerance to stress index	STI = (Dws × Dwp)/Dwp
relative growth rate	RGR = (ln Dw2 – ln Dw1)/(t2 – t1)
water use efficiency	WUE_p_ = Dw2/water used^b^
relative water content	RWC = (Wf – Wd)/(Wt – Wd)

a Dws, Dwp, Dw̅s, and Dw̅p represent weight under stress, weight under nonstress for each treatment, and weight means in stress and nonstress conditions for all treatments, respectively. Dw1 and Dw2 indicate seedling dry weights at times t1 and t2 (t1 is the beginning and t2 is the end of the period studied), respectively. Wf, Wd, and Wt refer to fresh, dry, and turgor weights, respectively.

b Considering all water used over the experimental period.

### 2.6 Gas Exchange Measurements

Gas exchange analyses were carried out on the fully developed leaves (*N* = 30). Photosynthesis (Pn), stomatal conductance (Gs), and transpiration rate (E) were measured on the attached leaves using a portable infrared gas analyzer (LCPro, BioScientific Ltd., Hoddesdon, UK). Measurements were made at environment CO_2_ concentration, a photosynthetic photon flux density (PPFD) of 1000 μmol m^–2^ s^–1^ (optimized with a light curve), and a cuvette airflow of 500 mL min^–1^.

### 2.7 Proline Determinations

The proline concentration at each experimental time point was calculated as the average of six plants. Proline content was determined as described previously.^30^ The samples of 20–50 mg of dry tissue were ground and extracted with 4 mL of 3% 5-sulfosalicylic acid. The extraction was centrifuged at 15,000*g* for 30 min, and 2 μL was mixed with 2 mL of acid ninhydrin and incubated at 100 °C for 60 min. This reaction was stopped in an ice bath. After extraction with 4 mL of toluene, the absorbance of the organic phase was measured at 520 nm in an Aquarius CE7200 double-beam spectrophotometer (Cecil Instruments, Cambridge, England). The proline concentration was calculated from a standard curve and normalized to dry weight.

### 2.8 Mineral Concentration in Tomato Plants

For the analysis of macronutrients (Ca, K, Mg, and P) and micronutrients (Fe, Mn, Cu, and Zn), tomato leaf samples from each treatment were collected at the end of the water-deficit and rehydration experiments. Samples were dried at 80 °C and then ground using an IKA M20 mill. Samples were then kept in an oven at 105 °C for 5 h and then transferred to a desiccator for weighing. Five hundred milligrams of ground powder was taken from each tomato sample and, after conversion to ash, mineralized in a muffle furnace at 480 °C dry with 6 N HCl. The mineral content was determined with the Avio 500 ICP-OES (PerkinElmer) using a standard curve method. All measurements were carried out in triplicate.

### 2.9 Statistical Analyses

One-way ANOVA tests (Duncan’s post hoc) were applied to analyze the differences between treatments in all measures studied. All statistical studies were performed on the IBM-SPSS24 statistical package.

## 3 Results and Discussion

### 3.1 Chitosan-Enclosed MSB Enhanced Tolerance to Water Deficiency

**Figure 1:**
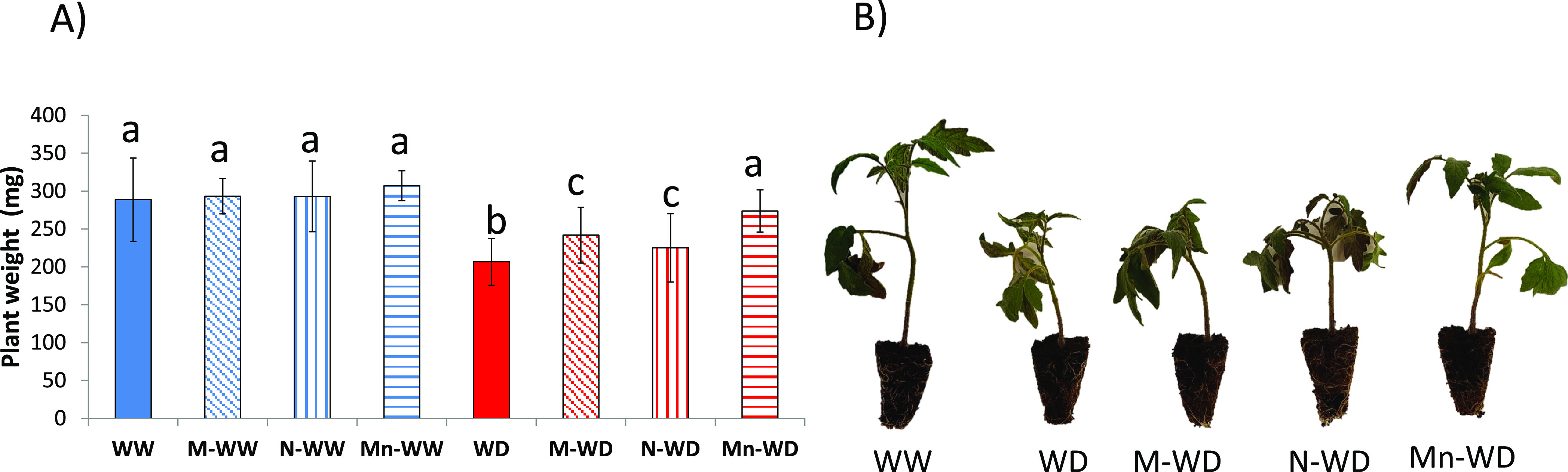
(A) Plant dry weight and (B) visual aspect of plants with water deficit compared to well-watered plants. Bars with the same letter show no significant differences (*p* < 0.05).

Plant resistance to water stress involves a variety of physiological, biochemical, and molecular responses that impact plant survival. Water-deficit stress can be defined as a situation in which the plant’s water potential and turgor are reduced to such an extent that normal functions are impaired. It is characterized by a reduction in water content, turgor, total water potential, wilting, and stomata closure and a decrease in cell enlargement and growth.^31^ Well-watered plants showed no significant differences between the different treatments (Figure 1A). Plants exposed to stress had a significant 30% reduction in plant growth after 7 days (Figure 1). Treatment with menadione sodium bisulfite (MSB) or empty nanoparticles (Nps) alone did increase plant tolerance, although the growth reduction was less than untreated plants (11 and 5%, respectively), consistent with previous reports on the use of MSB^16^ and chitosan^32^ (CHI) against water-deficit stress. Indeed, Mn further increased tolerance to the water deficit. The encapsulated compound significantly reduced weight loss by 18% compared to untreated plants, but no difference was shown with the well-watered plants. Moreover, the weight was also 7 and 13% higher than watered-deficit plants treated with MSB (M-WD) and watered-deficit plants treated with empty nanoparticles (N-WD), respectively (Figure 1A), and the protective effect is clearly visible (Figure 1B). There are some similar reports where encapsulation of an active ingredient improves the sole tolerance to water deficit in the compounds studied,^29,33^ showing how nanocapsules can act synergistically with Bs to protect the plant.

**Figure 2:**
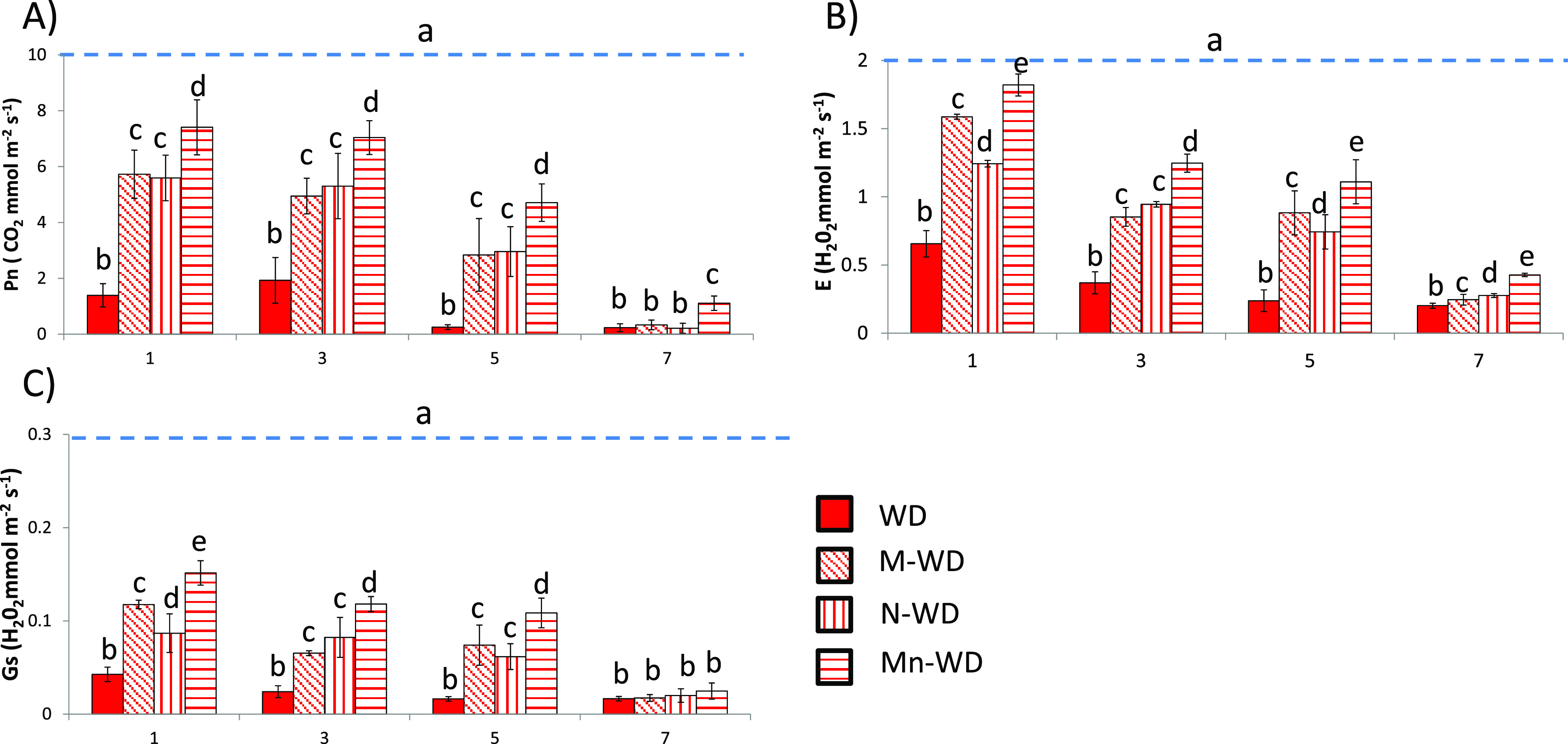
(A) Net photosynthesis, (B) evapotranspiration, and (C) stomatal conductance. The blue dashed line represents the values of well-watered plants (full values are displayed in the Supporting Information). Bars with the same letter show no significant differences (*p* < 0.05).

The immediate reaction of plants exposed to drought stress is the closing of stomata. However, closing stomata not only reduces water loss through transpiration but also CO_2_ and nutrient uptake, altering metabolic pathways such as photosynthesis.^34^ The values of Pn (Figure 2A), E (Figure 2B), and Gs (Figure 2C) decrease throughout the period studied, reaching the lowest value after 5 days of exposure to stress. The experiments of water-deficit plants treated with MSB (M-WD) and water-deficit plants treated with empty nanoparticles (N-WD) show a significant decrease in the mentioned parameters (Figure 2); however, the decrease in gas values was lower compared to the untreated plants, as described in previous reports where MSB^35^ and CHI^32^ protected photosynthesis from stress. However, Mn showed better gas exchange parameters with the lowest decrease in all measured parameters (Figure 2), which explains the increased weight of the plants after water deficit (Figure 1A). This synergistic behavior has already been described in the literature and shows that encapsulation of active ingredients can enhance the effect of MSB more than the effect by it alone.^36^

The differences between treatments are particularly clear when we study the data using different growth and stress indices (Table 3). Water deficit affects plant growth after 4 days. Only water-deficit plants treated with MSB-loaded Nps (Mn-WD) were able to reduce this difference, and the decrease in growth is especially dramatic in water-deficit plants treated with empty nanoparticles (N-WD), where there was no protection in 0 to 4 day treatments. A similar behavior is shown with WUE; during the first period, all plants subjected to a water deficit were higher, but the plants not treated with MSB had lower levels of WUE compared to their well-watered counterparts in the period of 4 to 7 days, which indicates that plants are unable to acclimate adequately to the imposed stress.^37^ Drops in RGR and WUE are especially interesting in water-deficit plants treated with MSB (M-WD); MSB alone is capable of increasing tolerance more so during the first part of the experiment, and then the effect begins to wane, comparable to the study of Venegas-Molina et al.^16^ Taking into account that a lower SSI indicates a higher tolerance against stress,^38^ such is clearly lower in water-deficit plants treated with MSB (M-WD) and, specially, watered-deficit plants treated with MSB-loaded Nps (Mn-WD), showing the enhanced tolerance given by the enclosed Bs. It is worth noting that the MSB reaches higher levels of STI. This index is positively correlated with higher yields under stress,^39^ showing how the MSB, particularly when encapsulated, is an excellent way to increase plant tolerance against drought.

**Table 3:** Stress Index Studied in the Water-Deficit Period^a^

	RGR_0 to 4 days_	RGR_4 to 7 days_	WUE_0 to 4 days_	WUE_4 to 7 days_	SSI_7 days_	STI_7 days_
WW	0.17	0.13	2.4	3.2		
M-WW	0.18	0.12	2.5	2.9		
N-WW	0.16	0.15	2.2	3.5		
Mn-WW	0.19	0.13	2.8	3.4		
WD	0.14	0.05	3.9	2.1	1.4	0.71
M-WD	0.16	0.09	4.4	3.8	0.88	0.82
N-WD	0.18	0.03	5.2	1.6	1.16	0.76
Mn-WD	0.17	0.11	4.9	5.1	0.54	0.89

a RGR, relative growth rate; WUE, water use efficiency; SSI, stress susceptibility index; STI, stress tolerance index.

**Figure 3:**
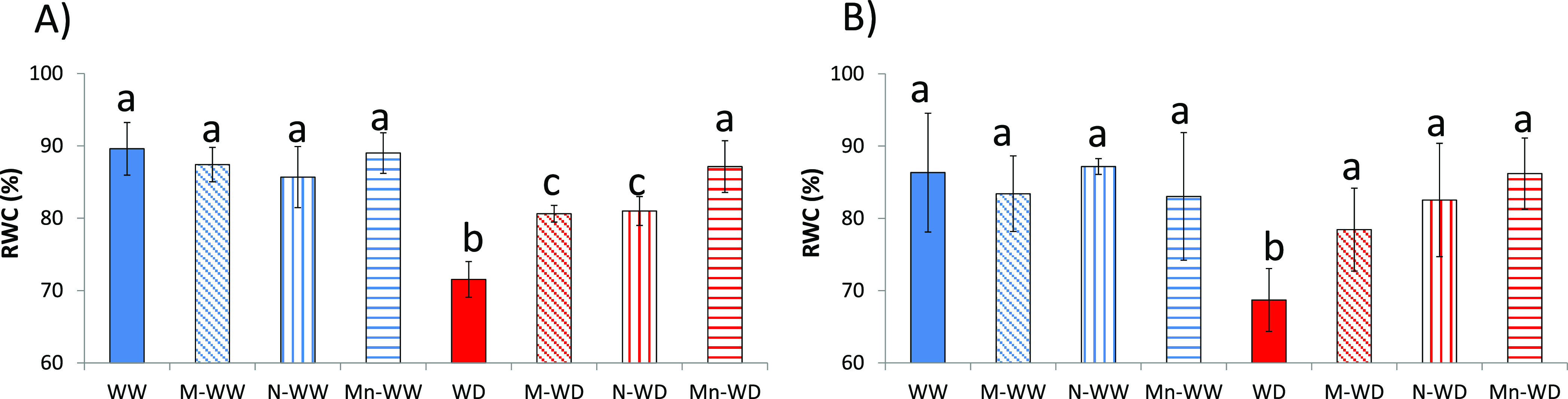
RWC, relative water content. After (A) 4 days and (B) 7 days of stress imposition. Bars with the same letter show no significant differences (*p* < 0.05).

**Figure 4:**
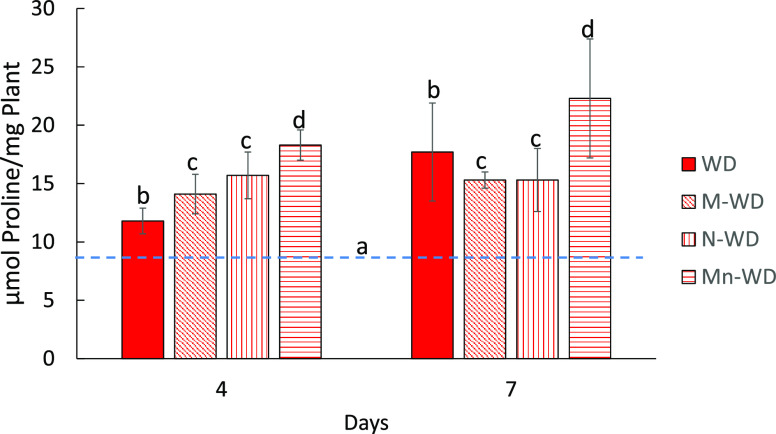
Proline concentration after (A) 4 days and (B) 7 days of stress imposition. The blue dashed line represents the values of well-watered plants (full values are displayed in the Supporting Information). Bars with the same letter show no significant differences (*p* < 0.05).

RWC is an important indicator of water status in plants, reflecting the balance between water supply and transpiration rate.^40^ WD plants exposed to stress had a significant decrease in RWC after 4 days (Figure 3A), and water-deficit plants treated with MSB (M-WD) and water-deficit plants treated with empty nanoparticles (N-WD) clearly improved water retention; however, water-deficit plants treated with MSB-loaded Nps (Mn-WD) were not significantly different from well-watered plants. After 7 days of stress imposition, WD continues with low RWC, but water-deficit plants treated with MSB (M-WD) and water-deficit plants treated with empty nanoparticles (N-WD) reach nonsignificant RWC levels compared to well-watered plants, like water-deficit plants treated with MSB-loaded Nps (Mn-WD). Plants are able to increase active osmotic compounds to counteract the negative pressure exerted by dry soil.^10^ Proline is probably one of the most common metabolites that plants use to perform osmotic adjustment,^14^ demonstrating that proline-overaccumulating plants are capable of increasing growth under hyperosmotic stress.^41^ Proline biosynthesis requires high amounts of NADPH and ATP and contributes to maintaining a low NADPH:NADP+ ratio in the chloroplast, thereby reducing photoinhibition and photosynthetic apparatus damage.^14^ WD plants subjected to stress clearly increase the amount of this amino acid after 4 days and even higher after 7 days (Figure 4). Water-deficit plants treated with MSB (M-WD) and water-deficit plants treated with empty nanoparticles (N-WD) had higher proline concentrations compared to WD, and this likely explains the RWC behavior previously discussed (Figure 3). The proline content of water-deficit plants treated with MSB-loaded Nps (Mn-WD) (Figure 4) is consistent with the RWC values, again demonstrating the synergy caused by the Bs encapsulation.

The plant ionome is defined as the mineral composition changes of the plant in response to physiological and environmental stimuli;^42^ as an example under water deficit, plant transpiration decreases and thus plays an important role in plant nutrient uptake.^43^ Water deficit leads to significant changes in nutrient uptake in tomato,^44^ which is clearly shown by our results where plants exposed to water deficit showed significant reductions in macronutrients (K^+^, Ca^2+^, and Mg^2+^) and micronutrients (Fe^2+^ and Cu^2+^) (Table 4). Water-deficit plants treated with empty nanoparticles (N-WD) showed the same behavior, except for Mg and Cu. However, water-deficit plants treated with MSB (M-WD) and water-deficit plants treated with MSB-loaded Nps (Mn-WD) showed significantly higher nutrient accumulation under water deficit than the other treatments, showing the protective effect of MSB on nutrient flux under stress conditions (Table 4). This correlates with the transpiration rate, which is higher in MSB-treated plants (Figure 2) and is consistent with other published results that MSB is able to prevent ion losses under osmotic stress by salt.^35^ Apart from the correlation with transpiration, these results are interesting because it is worth noting that water-deficit stress in plants leads to a reduction in nutrient uptake, which may have implications for human health.^45^ Treatments with MSB-loaded Nps could be an interesting alternative to consider under field conditions, aiming to address water scarcity issues due to climate change and its effects on human nutrition.

**Table 4:** Plant Ionome under Water Deficit^a^

	%				ppm			
	K	P	Ca	Mg	Fe	Mn	Cu	Zn
WW	2.1 ± 0.01a	0.2 ± 0.01a	1.7 ± 0.03a	0.5 ± 0.02a	95 ± 4a	79 ± 8.9a	19 ± 0.9a	32 ± 1.2a
M-WW	2.4 ± 0.01b	0.3 ± 0.02b	2.1 ± 0.05b	0.6 ± 0.05a	104 ± 5a	93 ± 9.3a	20 ± 0.4a	35 ± 1.3a
N-WW	2.0 ± 0.02c	0.3 ± 0.03b	1.7 ± 0.04a	0.5 ± 0.07a	89 ± 3a	73 ± 6.6a	18 ± 0.1a	29 ± 0.6b
Mn-WW	2.2 ± 0.01d	0.3 ± 0.01b	2.0 ± 0.01b	0.6 ± 0.03a	110 ± 8a	84 ± 4.4a	19 ± 1.1a	36 ± 0.8c
WD	1.6 ± 0.01e	0.2 ± 0.01a	1.4 ± 0.01c	0.1 ± 0.02b	63 ± 3b	65 ± 5.8a	15 ± 1b	39 ± 0.5d
M-WD	1.8 ± 0.01f	0.2 ± 0.04a	1.6 ± 0.05a	0.5 ± 0.01a	76 ± 4c	78 ± 7.2a	16 ± 0.6b	39 ± 0.3d
N-WD	1.6 ± 0.01e	0.2 ± 0.01a	1.3 ± 0.01c	0.4 ± 0.02a	69 ± 2b	68 ± 9.1a	19 ± 0.5a	31 ± 0.4b
Mn-WD	1.9 ± 0.01g	0.3 ± 0.02b	1.7 ± 0.02a	0.5 ± 0.01a	77 ± 4c	76 ± 4.3a	17 ± 0.6a	37 ± 0.6d

a Results with the same letter show no significant differences (*p* < 0.05).

MSB treatment has previously been described as a treatment that can increase growth under water-deficit stress.^16^ Instead, MSB can accelerate the accumulation of proline and abscisic acid, leading to better water and gas exchange processes under osmotic stress conditions.^35^ Overall, our results revealed that plants treated with MSB-loaded Nps could perform better during the water deficit period, which finds a good correlation with the few reports regarding the encapsulation of biostimulants using chitosan.^26,46^ The results indicate the synergistic effect shown after encapsulation, which is probably due to a better MSB assimilation of the plant, possibly enhanced for an efficient interaction taking place between chitosan and vegetal tissues, increasing the MSB protective effect over the entire water-deficit stress period. However, more research is needed to clarify how the encapsulated treatment can increase the effect compared to the use of the free compound.

### 3.2 Chitosan-Enclosed MSB Enhanced Plant Recovery after Rehydration

**Figure 5:**
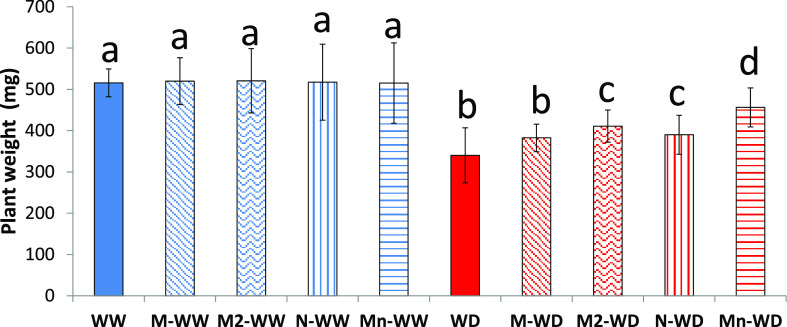
Plant dry weight. Bars with the same letter show no significant differences (*p* < 0.05).

Plant recovery after rehydration is an essential trait for plant survival and reflects the balance between reconstruction of damaged structures and adequate metabolism restoration.^47^ After the first experimental period, all plants were watered at full field capacity. Again, all treatments assayed did not show differences between the well-watered groups. The drought period clearly affected plant growth as is demonstrated in WD plants after the rehydration period (Figure 5), which had a 34% growth reduction compared to WW plants, slightly higher in the first 7 days during the water deficit assay. Water-deficit plants treated with MSB (M-WD) were not significantly different than the WD plants, 26% lower than the WW plants, consistent with Venegas-Molina et al.’s^16^ findings that MSB treatment losses efficacy after 1 week. However, a second treatment of MSB slightly, but significantly, increased the weight in comparison with WD and water-deficit plants treated with MSB (M-WD), again demonstrating that the MSB treatment efficacy decreases, most likely because it is easily degradable due to its thermal and pH sensibility.^17^ Results for water-deficit plants treated with empty nanoparticles (N-WD) continue to be significantly higher than those for WD; however, again, the best results are reached with the encapsulating MSB, although those plants had significantly lower weights as compared to the well-watered plants. That demonstrates again the synergistic effect of the drug encapsulation, which gives interesting properties for further exploration under field conditions.

**Figure 6:**
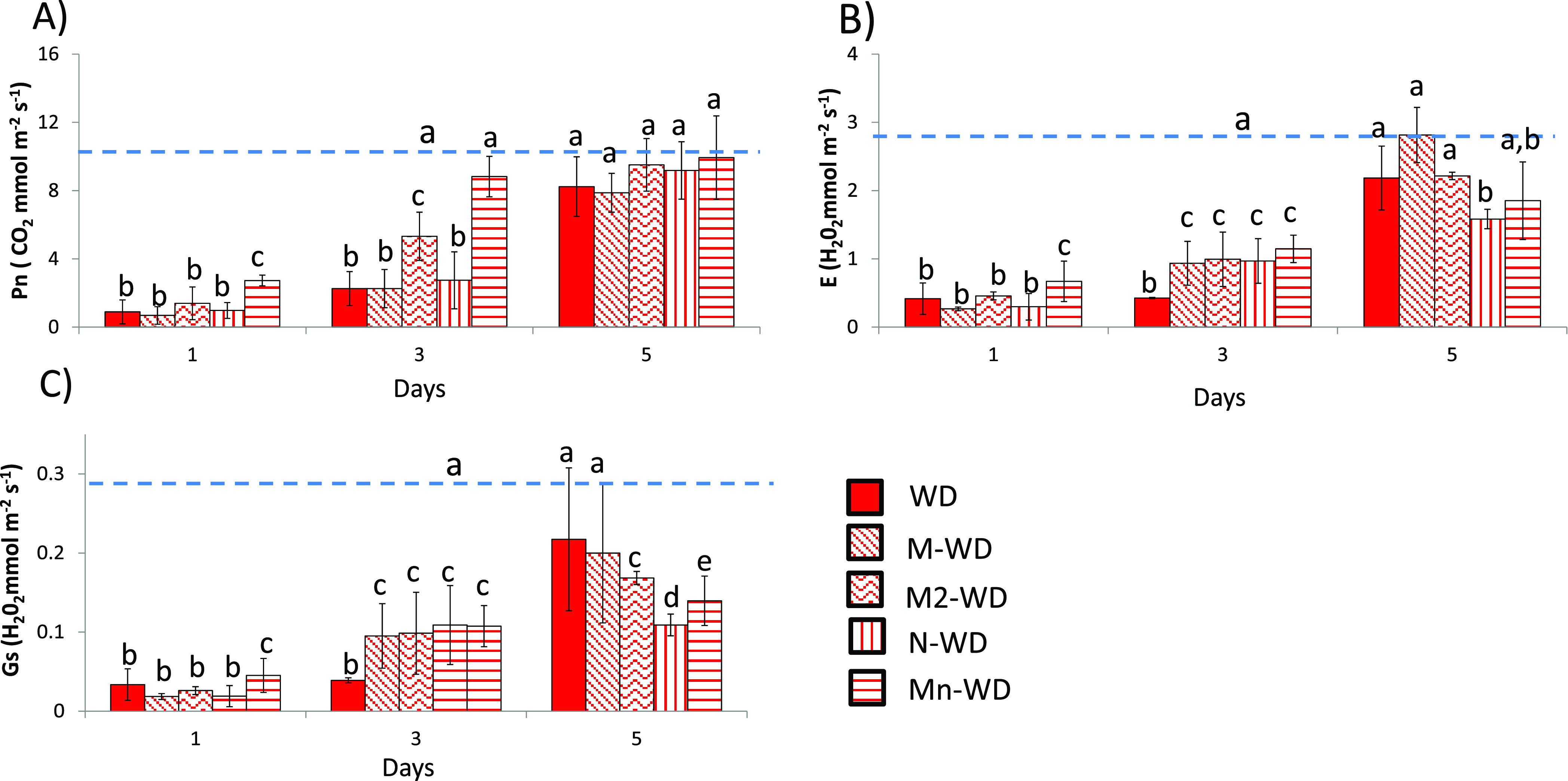
(A) Net photosynthesis, (B) evapotranspiration, and (C) stomatal conductance. The blue dashed line represents the values of well-watered plants (full values are displayed in the Supporting Information). Bars with the same letter show no significant differences (*p* < 0.05).

The leaf gas exchange parameters of plants subjected to water deficit stress during rehydration tend to reach the well-watered threshold^48^ (Figure 6). However, net photosynthesis only recovered to well-watered values after 5 days in WD, well-watered plants treated with MSB (M-WD), M2-WD, and water-deficit plants treated with empty nanoparticles (N-WD) (Figure 6A). Interestingly, the plants subjected to a second treatment had more significant values after only 3 days of rehydration, but only the water-deficit plants treated with MSB-loaded Nps (Mn-WD) reached the well-watered threshold after 3 days. It is worth noting that the M2-WD plants and water-deficit plants treated with MSB-loaded Nps (Mn-WD) had lower significant values of stomatal conductance (Figure 6C), and this behavior is consistent with our previous study,^35^ where the MSB can recover photosynthesis quicker under mild salt stress, likely due to MSB’s clear impact on abscisic acid accumulation,^35^ conferring better stomatal conductance control.

**Figure 7:**
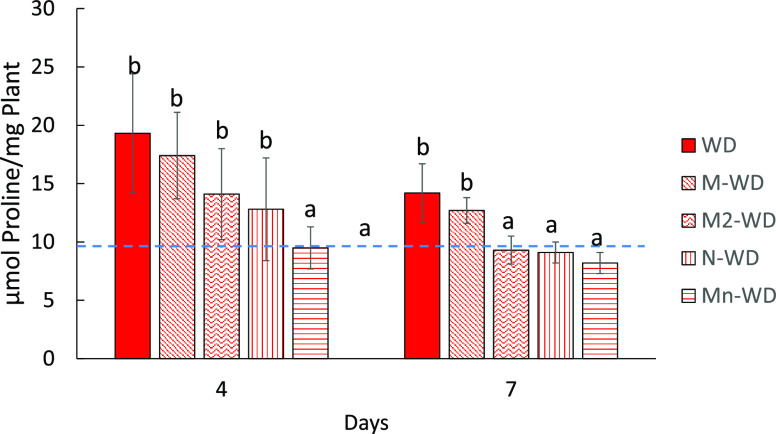
Proline concentration after (A) 4 days and (B) 7 days of rewatering began. The blue dashed line represents the values of well-watered plants (full values are displayed in the Supporting Information). Bars with the same letter show no significant differences (*p* < 0.05).

Plants, after a water deficit period, had increased (Figure 4) to counteract the negative osmotic pressure exerted by water soil deprivation.^10^ However, after the stress, the proline concentration decreased, allowing the system to return to “normal” levels in plant tissues.^49^ In this regard, our results suggest that all treatments lower proline levels after the onset of rehydration (Figure 7). However, WD and water-deficit plants treated with MSB (M-WD) did not reach the WW concentration, and the drop in proline levels is clear. Again, the second treatment with MSB in M2-WD plants had similar values to the water-deficit plants treated with empty nanoparticles (N-WD), reaching the WW proline concentration after 7 days of rewatering. Interestingly, water-deficit plants treated with MSB-loaded Nps (Mn-WD) significantly decreased their proline levels only after 4 days of rewatering. After a stress period, proline is catabolized in the mitochondria, supporting oxidative respiration with energy to resume growth after stress. Indeed, complete oxidation of proline would yield 30 ATP molecules. Therefore, proline reserves are valuable not only in osmotic adjustment during acclimation but also in facilitating recuperation after stress.^50^ Our results support this idea, because the plants treated with encapsulated MSB reduced proline levels quicker, showing that plants are not only capable of better adjusting to osmotic stress, but they are also capable of resuming growth faster with better stress performance.

In conclusion, CHI Nps have been shown in previous reports to be a good carrier for plant treatments, capable of enhancing the beneficial effects of the compounds in agriculture,^24^ as it is readily taken up by plant tissues.^25^ In this respect, our results clearly show how the plants treated with MSB encapsulated had higher plant tolerance compared to ones treated with free MSB. In addition, we would like to point out that biostimulants are sometimes expensive and require continuous treatments to achieve good results.^51^ We chose MSB for our experiments mainly for two reasons: (i) it is an interesting compound that can ameliorate a wide range of biotic and abiotic stresses^15^ and, of course, drought stress^16^ and (ii) the beneficial effect of MSB against water deficit is limited to 1 week. Here, we clearly demonstrated how MSB-loaded Nps can delay the necessary MSB treatment to cope with the stress by at least 1 week. In our opinion, this opens the nanoencapsulation (NE) possibilities not only to increase the protective behavior of the biostimulants, but we have also shown here that NE increases the durability of the compound by extending the time between treatments, which is an interesting avenue for further research under field conditions.

## Supporting Information Available

Table S1: full gas exchange measurements from Figures 2 and 6; Table S2: full proline concentration measurements from Figures 4 and 7; Figure S1: measurement of particle size (PDF)
Growth datasets (XLSX)

## Abbreviations

MSB: menadione sodium bisulfite
Bs: biostimulant
NE: nanoencapsulation
Nps: nanoparticles
CHI: chitosan
TPP: sodium tripolyphosphate
AcOH: glacial acetic acid
NaOH: sodium hydroxide
