# Supplementary material for: Chitosan-Enclosed Menadione Sodium Bisulfite as an Environmentally Friendly Alternative to Enhance Biostimulant Properties against Drought
Source: J Agric Food Chem. 2023 Feb 9;71(7):3192–200. doi: 10.1021/acs.jafc.2c07927 (PMC9951248; doi:10.1021/acs.jafc.2c07927)
Supplement: Supplementary file 1 — jf2c07927_si_001.pdf [file jf2c07927_si_001.pdf]

Supporting information for

Chitosan-enclosed menadione sodium bisulfite as an environmentally friendly alternative to enhance biostimulant properties against drought

David Jiménez-Arias[1*], Sebastian Bonardd[2,3], Sarai Morales-Sierra[4], Miguel Â. Almeida Pinheiro de Carvalho[1,5*] and David Díaz Díaz[2,3,6*]

1. ISOPlexis, Center for Sustainable Agriculture and Food Technology, Madeira University, Campus Universitário da Penteada, 9020-105 Funchal, Madeira, Portugal

2. Departamento de Química Orgánica, Universidad de la Laguna, Avda. Astrofísico Francisco Sánchez 3, La Laguna, 38206, Tenerife, Spain

3. Instituto Universitario de Bio-Orgánica Antonio González, Universidad de la Laguna, Avda. Astrofísico Francisco Sánchez 2, La Laguna, 38206, Tenerife, Spain

4. Grupo de Biología Vegetal Aplicada, Departamento de Botánica, Ecología y Fisiología Vegetal-Facultad de Farmacia, Universidad de La Laguna, Avenida. Astrofísico Francisco Sánchez s/n,38071 La Laguna, Tenerife, Canary Islands, Spain

5. CiTAB, Centre for the Research and Technology of Agroenvironmental and Biological Sciences. University of Trás-os-Montes and Alto Douro. Quinta dos Prados. 5000-801 Vila Real, Portugal.

6. Institute of Organic Chemistry, Faculty of Chemistry and Pharmacy, Regensburg University, Regensburg

*Corresponding author: david.j.a1983@gmail.com; miguel.carvalho@staff.uma.pt; ddiazdiaz@ull.edu.es

Table S1. Gas Exchange full measurements from figure 2 and 6.

Stomatal Conductance (H_20_2 mmol m^{-2} s^{-1})							
	1	3 ±	5 ±	7	1	3	5
WW	0.3 ± 0.03	0.3 ± 0.02	0.3 ± 0.005	0.3 ± 0.02	0.3 ± 0.04	0.2 ± 0.03	0.4 ± 0.005
M-WW	0.3 ± 0.01	0.3 ± 0.02	0.3 ± 0.008	0.3 ± 0.01	0.2 ± 0.02	0.2 ± 0.02	0.4 ± 0.009
M_2-WW	-	-	-	-	0.3 ± 0.03	0.2 ± 0.02	0.4 ± 0.008
N-WW	0.3 ± 0.01	0.3 ± 0.01	0.4 ± 0.02	0.3 ± 0.01	0.3 ± 0.01	0.2 ± 0.01	0.2 ± 0.004
Mn-WW	0.2 ± 0.06	0.2 ± 0.01	0.2 ± 0.004	0.2 ± 0.03	0.2 ± 0.06	0.1 ± 0.03	0.4 ± 0.006
WD	0.04 ± 0.007	0.02 ±0.006	0.02 ± 0.002	0.02 ± 0.002	0.03 ± 0.01	0.04 ± 0.003	0.2 ± 0.09
M-WD	0.12 ± 0.004	0.07 ± 0.002	0.07 ± 0.02	0.02 ±0.004	0.02 ± 0.003	0.09 ± 0.04	0.2 ± 0.08
M_2-WD	-	-	-	-	0.03 ± 0.004	0.09 ± 0.05	0.17 ± 0.008
N-WD	0.09 ± 0.02	0.08 ± 0.21	0.06 ± 0.01	0.02 ± 0.007	0.02 ± 0.01	0.11 ± 0.05	0.1 ± 0.01
Mn-WD	0.15 ± 0.01	0.12 ± 0.008	0.11 ± 0.01	0.03 ± 0.008	0.04 ± 0.02	0.11 ± 0.03	0.1 ± 0.03
	Water Deficit				Rehydration		

Evapotranspiration (H_20_2mmol m^{-2} s^{-1})							
	1	3	5	7	1	3	5
WW	3.0 ± 0.3	1.2 ± 0.09	1.8 ± 0.005	2.0 ± 0.02	3.1 ± 0.3	2.8 ± 0.1	2.9 ± 0.4
M-WW	2.3 ± 0.09	1.8 ± 0.6	2.3 ± 0.008	2.1 ± 0.01	2.4 ± 0.05	1.7 ± 0.4	2.5 ± 0.02
M_2-WW	-	-	-	-	2.8 ± 0.2	1.4 ± 0.3	2.2 ± 0.2
N-WW	3.2 ± 0.2	1.8 ± 0.5	2.5 ± 0.02	2.5 ± 0.01	3.3 ± 0.2	1.6 ± 0.7	1.7 ± 0.6
Mn-WW	2 ± 0.3	1.7 ± 0.7	1.6 ± 0.004	1.7 ± 0.03	2.1 ± 0.4	1.1 ± 0.1	2.1 ± 0.4
WD	0.7 ± 0.09	0.4 ± 0.08	0.2 ± 0.01	0.2 ± 0.01	0.4 ± 0.2	0.4 ± 0.009	2.2 ± 0.4
M-WD	1.6 ± 0.11	0.9 ± 0.06	0.9 ± 0.07	0.2 ± 0.02	0.2 ± 0.03	0.9 ± 0.3	2.8 ± 0.4
M_2-WD	-	-	-	-	0.4 ± 0.06	0.9 ± 0.4	2.2 ± 0.05
N-WD	1.2 ± 0.02	0.9 ± 0.02	0.7 ± 0.1	0.3 ± 0.01	0.2 ± 0.2	0.9 ± 0.3	1.6 ± 0.1
Mn-WD	1.8 ± 0.08	1.2 ± 0.07	1.1 ± 0.1	0.4 ± 0.01	0.6 ± 0.3	1.1 ± 0.2	1.8 ± 0.6
	Water Deficit				Rehydration		

Net Photosyntesis (CO_2 mmol m^{-2} s^{-1})							
	1	3	5	7	1	3	5
WW	11.3 ± 1.6	9.8 ± 1.2	8.8 ± 2.3	10 ± 1.7	11.9 ± 1.6	9.3 ± 1.2	9.7 ± 2.5
M-WW	10.0 ± 1.4	10.6 ± 2.9	9.3 ± 0.9	10.1 ± 1.7	10.5 ± 1.5	10.1 ± 2.9	10.2 ± 0.9
M_2-WW	-	-	-	-	11.2 ± 1.6	9.7 ± 2	10.0 ± 1.7
N-WW	10.8 ± 1.7	11.1 ± 3.1	9.1 ± 0.5	10.4 ± 1.7	11.4 ± 1.8	9.1 ± 2.3	8.1 ± 1.6
Mn-WW	9.8 ± 1.2	9.6 ± 2.3	7.3 ± 2.1	9.9 ± 1.6	10.4 ± 1.2	8.9 ± 1.1	10.7 ± 2.7
WD	1.3 ± 0.4	1.9 ± 0.8	0.2 ± 0.08	0.2 ± 0.1	0. 9 ± 0.7	2.26 ± 0.9	8.2 ± 1.7
M-WD	5.7 ± 0.9	4.9 ± 0.6	2.8 ± 1.3	0.3 ± 0.2	0.68 ± 0.7	2.26 ± 1.1	7.8 ± 1.1
M_2-WD	-	-	-	-	1.3 ± 0.9	5.3 ± 1.4	9.5 ± 1.5
N-WD	5.5 ± 0.8	5.3 ± 1.1	2.9 ± 0.9	0.2 ± 0.2	0.9 ± 0.5	2.7 ± 1.7	9.2 ± 1.7
Mn-WD	7.4 ± 0.9	7.0 ± 0.6	4.7 ± 0.7	1.1 ± 0.3	2.7 ± 0.3	8.8 ± 1.2	9.9 ± 2.4
	Water Deficit				Rehydration		

Table S2. Proline concentration full measurements from figure 4 and 7.

µmol Proline/mg plant				
	4 days	7 days	4 days	7 days
WW	10,7 ± 1.5	8.8 ± 3.1	8.9 ± 0.8	9.2 ± 0.8
M-WW	12,2 ± 1.7	8.8 ± 2.2	8.9 ± 1.7	9 ± 0.7
M_2-WW	-	-	12,1 ± 1.3	10.2 ± 1.1
N-WW	10,8 ± 0.8	9.1 ± 3.8	10.1 ± 2.6	10.4 ±1.2
Mn-WW	11,6 ± 1.1	9.1 ± 2	10.8 ± 2.2	8.9 ± 2.5
WD	11,8 ± 1.1	17.7 ± 4.2	19,3 ± 5.1	14.2 ± 2.5
M-WD	14,1 ± 1.7	15.3 ± 0.7	17,4 ± 3.7	12.7 ± 1.1
M_2-WD	-	-	14,1 ± 3.9	9.3 ± 1.2
N-WD	15,7 ± 2	15.3 ± 2.7	12,8 ± 4.4	9.1 ± 0.9
Mn-WD	18,3 ± 1.3	22.3 ± 5.1	9,5 ± 1.8	8.2 ± 0.9
	Water deficit		Rehydration	

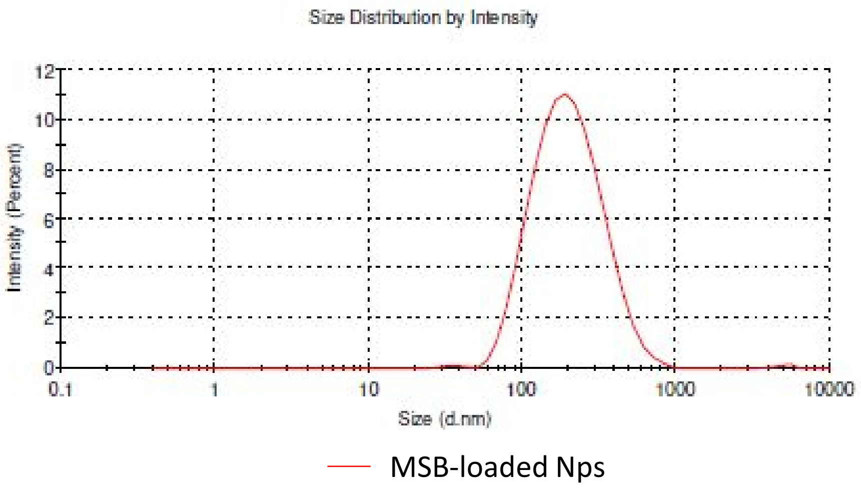

Figure S1. Measurement of particle size. The red bar represents the average of three individual measurements.
